# Detection and quantification of the giant protein titin by SDS-agarose gel electrophoresis

**DOI:** 10.1016/j.mex.2017.09.007

**Published:** 2017-10-10

**Authors:** Chaoqun Zhu, Wei Guo

**Affiliations:** Animal Science, University of Wyoming, Laramie, WY 82071, USA

**Keywords:** Large protein detection and quantification, Titin protein electrophoresis, Alternative splicing, Titin isoforms, Striated muscles

## Abstract

Titin, a giant sarcomeric protein, is involved in the generation of passive tension during muscle contraction, assembly and stability of the sarcomere in striated muscles. Titin gene produces numerous titin protein isoforms with different sizes (∼3–4 MDa) resulting from alternative splicing. To study titin and titin isoform changes under disease conditions, the method to detect and quantify titin protein isoforms is needed. The method reported here is a 1% vertical SDS-agarose gel electrophoresis system that can solubilize, detect and quantify various titin isoform sizes. Sodium dodecyl sulfate (SDS)-agarose gel electrophoresis is an important tool in revealing the size and quantity of giant proteins in the sarcomere. In this method article, heart tissues were dissolved in urea-thiourea-glycerol sample buffer. Muscle proteins were resolved on 1% SDS-agarose gels that were silver-stained subsequently. Titin isoform bands with different sizes were separated on the gel. At the end, we also validated the method for large protein detection. Our results indicated that this electrophoresis method is efficient to study the transitions in titin isoforms.

• This method provides efficient protein extraction with urea-thiourea-glycerol buffer from hard tissues such as striated muscles
• This method provides an efficient way to separate large proteins over 500 kDa
• Combining with silver staining, our method can detect large protein isoforms and quantify the separated protein bands.

## Graphical abstract

**Figure:**
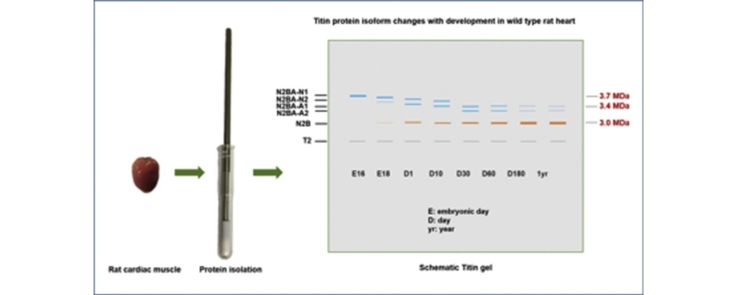

## Method details

Gel electrophoresis has been used extensively to determine the size and quantity of proteins. Muscle tissues such as cardiac and skeletal commonly express a variety of giant proteins (larger than 0.5 MDa), which play important roles in muscle structure and function. Titin, also called connectin, is the largest known sarcomere protein. In the sarcomere, titin plays a critical role in maintaining structural integrity and developing passive tension with stretch [1]. Titin can thus be viewed as a molecular spring in striated muscle. Titin gene undergoes alternative splicing, and generates numerous isoforms in the heart [2]. The size of different titin isoforms ranges from ∼3 to 4.2 MDa (theoretically 4.2 MDa is full size only when all exons are expressed which has never been detected by SDS-agarose gel so far) in the heart [3]. Titin N2B isoform is produced from a single splicing pathway with a size of approximately 3.0 MDa. N2BA isoforms are produced from multiple splicing pathways, and detectable N2BA isoforms are N2BA-A1 (adult form), A2 (adult form), N1 (embryonic and neonatal form) and N2 (embryonic and neonatal form) with sizes of about 3.4, 3.2, 3.7 and 3.6 MDa respectively [2], [4]. Recently, it has been reported that titin gene splicing is mainly regulated by RNA binding protein 20 (RBM20). In RBM20 knockout rat heart, a new N2BA isoform named N2BA-G with a size of approximately 3.9 MDa has only been expressed [5]. Cardiac titin isoforms alteration has been identified and associated with human heart failure [6], [7]. Gel electrophoresis is the simplest and most direct way to observe the alteration of titin isoforms during developmental and pathological changes. However, electrophoretic analysis of large proteins has been difficult to separate titin isoform proteins. In order to clearly and easily resolve various titin isoforms, SDS-agarose gels have been developed [8]. The present vertical SDS-agarose gel electrophoresis system has been modified and used as an efficient method for high-resolution separation of titin isoforms.

## Sample preparation

### Sample buffer preparation

The sample buffer was prepared specific for the striated muscle proteins, containing 8 M urea, 2 M thiourea, 30% glycerol, 3% SDS weight/volume (w/v), 75 mM DTT, 0.03% bromophenol blue, and 0.05 M Tris–HCl, pH 6.8. Urea and thiourea were added into a clean beaker and dissolved in glycerol. 10% of deionized water was added to the mixture to increase the dissolving rate (do not add too much water, the urea plus thiourea takes up over half of the final volume), and stirred gently in a hot plate until the solution was clean at room temperature. The heating temperature should be under 40 °C to avoid cyanate formation from heating urea. 10% w/v of a mixed bed resin (Fisher Rexyn 300 or Biorad AG 501-X8) was added to deionize urea buffer and remove cyanate, and the mixture was stirred at room temperature for 15 min. If you have a conductivity meter, the conductivity should be less than 5 μmhos. The mixture was filtered through filter paper to remove the resins. Tris-base and SDS were added, and the mixture was adjusted to pH 7.5 using HCl. Solid DTT was added and stirred till dissolved. The pH was continuously adjusted down to 6.8. Bromophenol blue was added and stirred till dissolved. The final volume was brought up by adding deionized water, and filtered through a Millex HA 0.45 or 0.22 μm filter (Millipore) to remove any fine particulate materiel. The filtered buffer was aliquoted into 1 mL or 2 mL microfuge vials, and stored at −20 °C for future use.

## Protein sample preparation

Cardiac tissues were dissected from wild type (Rbm20^+/+^, WT), heterozygous (Rbm20^+/−^, HT) and homozygous (Rbm20^−/−^, HM) rats and flash-frozen in liquid nitrogen. The Rbm20-deficient (HT and HM) rats were derived from a spontaneous mutant [5]. Rats used in the current work were crosses of Sprague-Dawley (SD) X Fisher 344 X Brown Norway (BN) (All strains were originally obtained from Harlan Sprague Dawley, Indianapolis, IN). For 1 mL of sample buffer, 10–15 mg tissue was used for protein extraction. Protease inhibitors addition were recommended during protein extraction. Tissues were homogenized using Dounce homogenizer on ice (0 °C), and protein samples were transferred to a 1.5 mL tube. Samples were then heated at 60 °C for 10 min, and subsequently centrifuged at 12,000 × *g* for 2 min. Supernatant was saved and used for gel loading or stored at −80 °C for future use. Protein samples are recommended 1:5 or 1:10 dilution with silver staining.

## Gel electrophoresis

### Agarose and acrylamide gel preparation

**Fig. 1:**
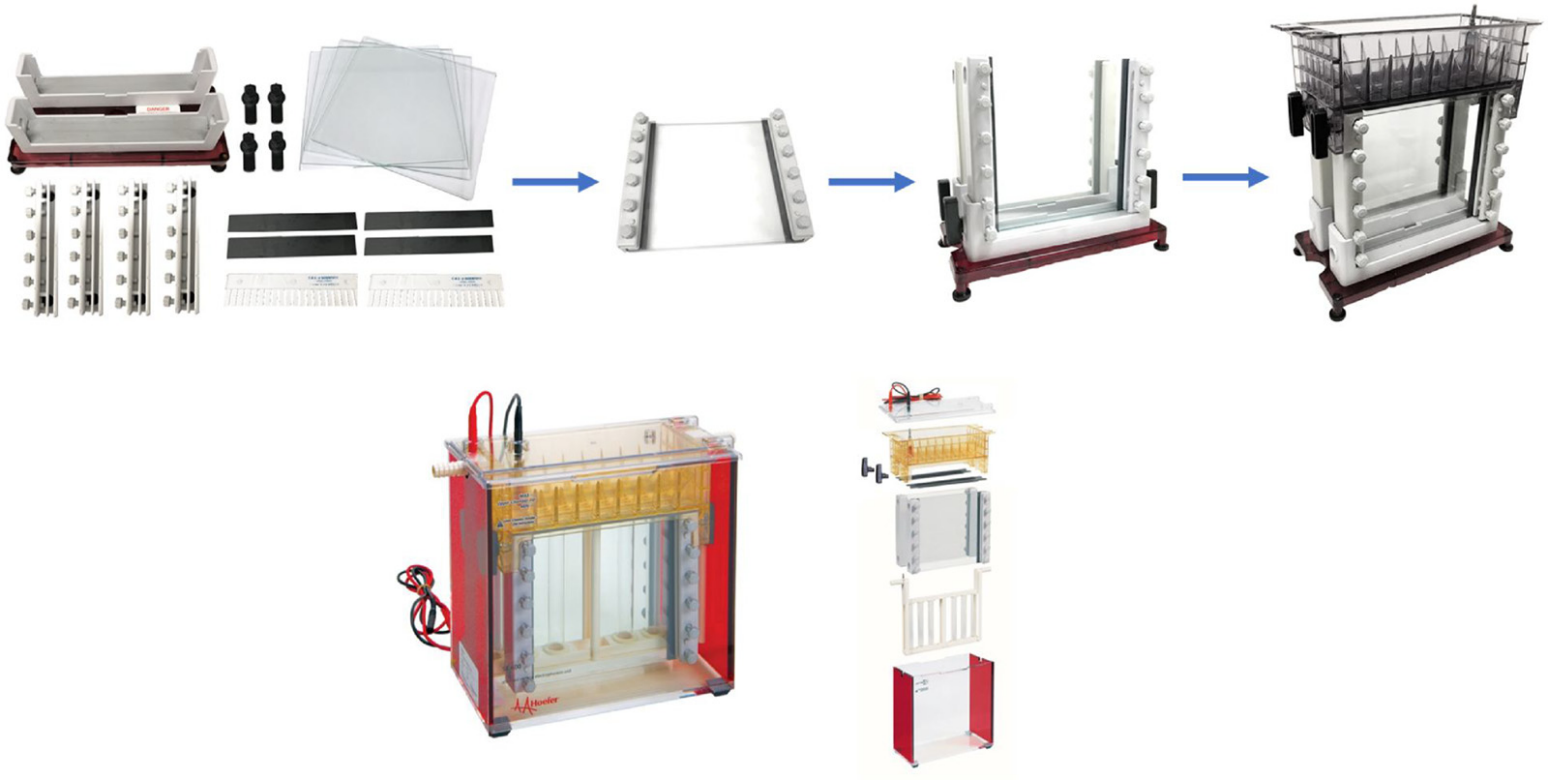
Gel electrophoresis apparatus and gel plate assembly.

The gel size was 1.5 mm × 16 cm × 18 cm. A Hoefer SE600 gel apparatus was used for gel preparation and electrophoresis (Fig. 1). The glass gel plates were thoroughly cleaned with soap and sprayed with ethanol, dried, and assembled using 1.5 mm spacers (Fig. 1). A 1 cm high acrylamide plug was needed to prevent agarose gel from sliding. The plug was consisted of 1.924 mL deionized water, 1.7 mL 50% glycerol, 2.12 mL 3 M Tris (pH 9.3), 2.72 mL acrylamide 40%, 24 μL 10% ammonium persulfate (APS), and 13 ul *N,N,N’,N’*-tetramethylethylenediamine (TEMED). 2.5 mL of the plug solution was added to each gel assembly to make a 1 cm plug. A small amount of water or ethanol was added on the top of each plug to even it and facilitate polymerization. The acrylamide plug was allowed to polymerize for 20–30 min. Water was drained off by inverting the gel unit and blotting off with paper towels after gel plug polymerization. Next, the gel assemblies were placed in an oven with over 60 °C and less than 80 °C for about 30 min preheating to prevent cracking of glass when pouring hot agarose in the next step. The resolving gel was the final composition which consists of 1% w/v Sea Kem Gold agarose (Biowhittaker Cell Biology Products, Walkersville, MD), 30% v/v glycerol, 20% 5× electrophoresis buffer (compositions see below). 0.8 g agarose powder was weighed out and put into a 500 mL beaker. In a 100 mL graduated cylinder, 48 mL 50% glycerol, 16 mL 5× electrophoresis buffer (0.25 M Tris-base, 1.92 M glycine, and 0.5% SDS, pH 8.5, no pH adjustment necessary) were added and the final volume of 80 mL was brought up by adding deionized water. The solution was mixed well and poured into the beaker with 0.8 g agarose and swirled to mix. The beaker was covered with plastic wrap and poked for a few holes. The beaker was heated with agarose and buffer in a microwave for about 3 min. The agarose and buffer mixture were heated until it began to boil and the agarose was completely dissolved. During the heating process, bubbles were produced, and the microwave was needed to be stopped for several times to prevent bubbles flushing out of the beaker. The last step of the gel making was pouring the agarose gel into the gel plates. The pre-warmed gel assembly was taken out of the oven and immediately the agarose was poured into the gel plates directly or using a pre-warmed 10 mL syringe (pre-warmed with gel assemblies mentioned above). About 40 mL was needed for each gel. Once the agarose was poured, the comb (1.5 mm thick and 20-lane) was inserted on the top of the gel. The agarose gel was allowed for 30–45 min to be cooled down and solidified at room temperature or shorter time in 4 °C. Gels could be used right away or could be stored overnight in 4 °C refrigerator.

Part of the figure was downloaded from http://www.hoeferinc.com.

### Sample loading and electrophoresis

The lower chamber contained 4 L of 1× buffer (50 mM Tris-base, 0.384 M glycine, and 0.1% SDS). The buffer was cooled to 4–6 °C with a circulating water bath. The upper chamber buffer was made by 600 mL cooled lower chamber buffer with the addition of 420 μL 2-mercaptoethanol. After preparation of electrophoresis buffer, the next step was sample loading. Combs were taken out of gels by bending them back and forth to detach from gel and slowly pulled them up. During comb removal, care must be taken not to break the wells. The gel wells were needed to be cleaned by pipetting with upper buffer several times to get rid of the sticky agarose residues and glycerol. This was a critical step for getting nice protein bands eventually. Gel wells were filled with upper buffer. Gel wells need be flushed again immediately before adding sample (the glycerol from the agarose tended to leak into the wells and would prevent the protein sample from settling to the bottom). Samples were loaded by placing tip all the way in the bottom (do not touch) of the well before releasing sample. The first and last lanes were skipped. The proper loading amount was between 2 and 5 μL. Once the samples were loaded, the upper chamber was put on assembly (Fig. 1). The upper chamber buffer was poured into the upper chamber from corners (do not pour buffer directly over wells). Place lid on, and run two gels at 30 mA constant current or 300–350 V constant voltage for 3 h. The blue dye will indicate the protein band migration. When the dye moves to the bottom of the acrylamide plug, the gel running should be done.

### Dissembling gels and fix solution preparation

After 3 h running, the gel plates were dissembled and the gel wells and acrylamide plug were removed and disposed. A spacer can be used to cut the gels, and also cut a small corner on one side of the gel to mark the order of lanes. The gels were then fixed in 50% methanol, 12% glacial acetic acid, and 5% w/v glycerol (500 mL for each gel) for at least 1 h with gentle shaking, up to 2 days in a sealed polypropylene container. The polypropylene containers were rinsed with concentrated nitric acid to remove mainly the precipitated silver to make thoroughly cleaned before use (Note: precipitated silver will cause high background if not cleaned out). The fixing solution were completely removed and gels were dried overnight in a 37 °C incubator. The overnight drying was important to obtain sharper and clear bands, and keep low staining background in the staining process.

### Staining

The silver staining method was used for agarose gels. All the wash steps and staining solution were prepared for 500 mL per gel and performed at room temperature with gentle shaking. After overnight drying, without changing the containers, the gels were washed 3 times with deionized water for at least 15 min each. The gels were then washed in potassium ferrocyanide (20 g/L) for 5 min and followed with deionized water 3 times for 5 min each time. The staining solution was prepared right prior to staining. Solution A contained 25 g of sodium carbonate in 500 mL of water (prepared in a 1 L beaker). Solution B contained 1 g silver nitrate, 1 g ammonium nitrate, 5 g tungstosilicic acid and 3 mL of 37% formaldehyde in 500 mL of deionized water. The solution B was slowly added to a stirring solution A just prior to staining. Gels were shaken in staining solution until bands appear (5–10 min, during staining process, the gel bands need be closely watched for not being over-stained). The staining solution was decanted and 500 mL of 1% v/v acetic acid was added to each gel, and shook the gel for 5 min to stop staining. The acetic acid was decanted and the gels were washed with deionized water for 5 min. Gels were dried between two sheets of cellophane paper (RPI #1080) with the addition of several drops of glycerol for one day.

## Method validation

### Gel well cleaning

**Fig. 2:**
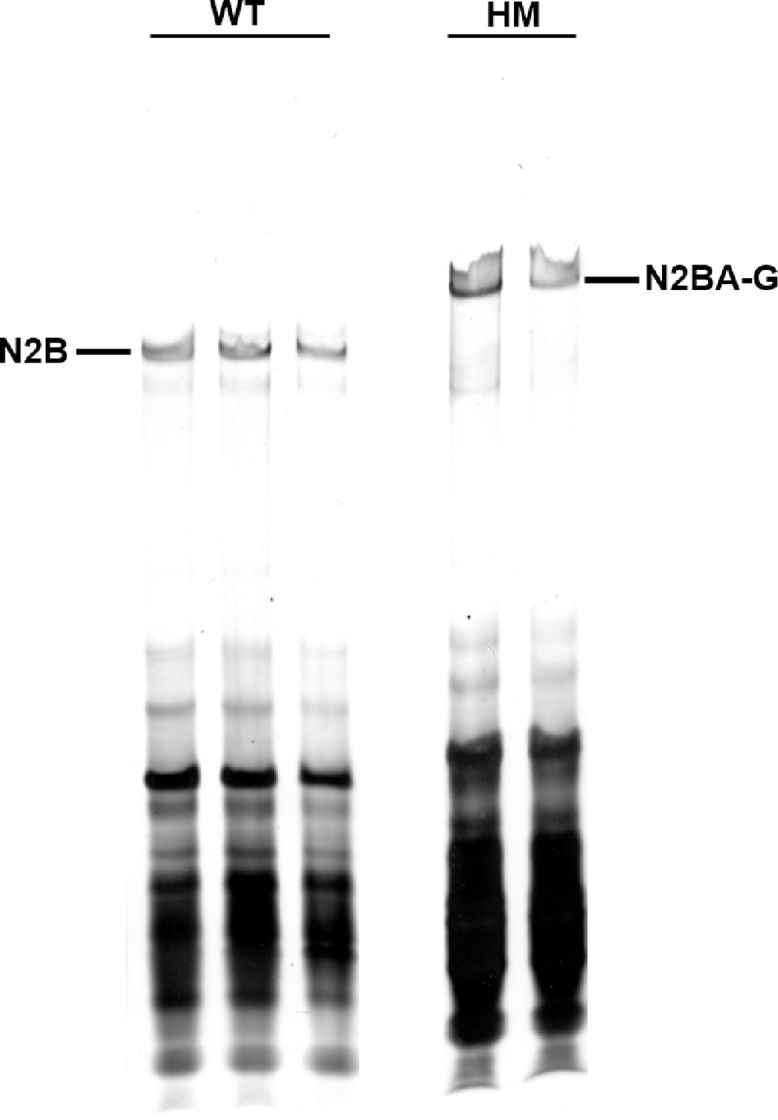
Not well-cleaned gel wells indicate fuzzy and not well-separated titin bands. WT, protein sample from heart tissues of wild type rats; HM, protein samples from homozygote RBM20 knockout rats. Titin proteins were adhered to the gel well, and different sized titin isoforms could not be separated from each other because of adhesion to the sticky gel well.

Gel wells cleaning by flushing with buffer is a critical and necessary step. Because the gel contains agarose and high concentration of glycerol, the gel wells are filled with sticky agarose residues and glycerol. Protein samples will be floating in the gel well instead of precipitating in the bottom if the gel wells are not completely cleaned. The protein bands will not show clear and sharp unless all the protein samples are nicely settling down to the bottom (Fig. 2).

### Sample dilution

**Fig. 3:**
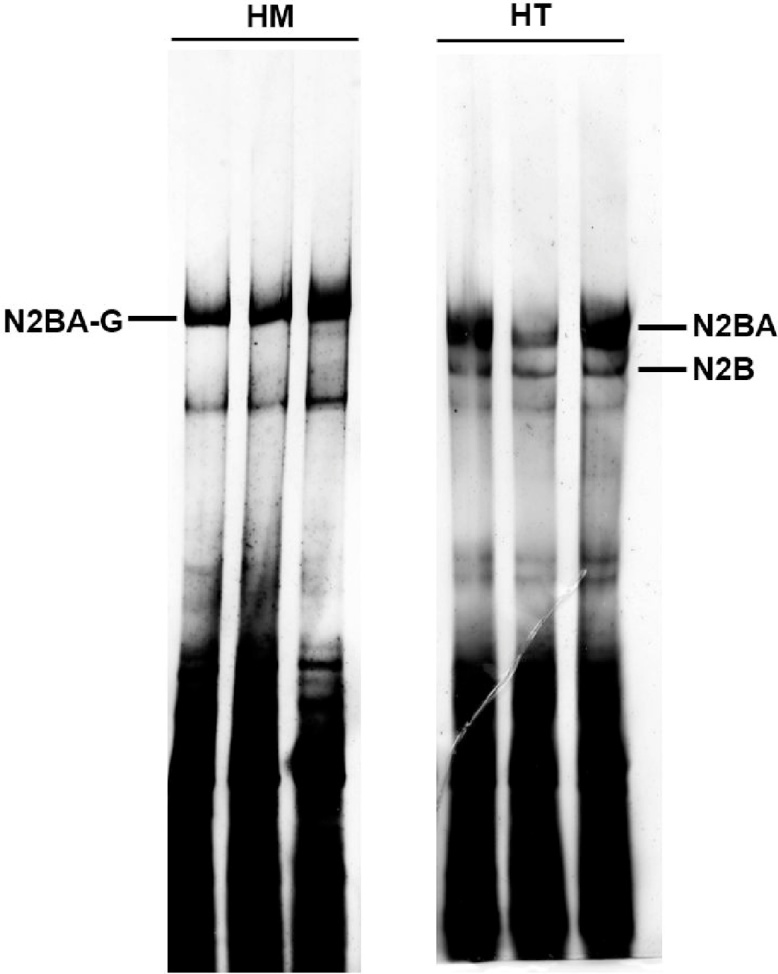
High protein sample concentration leads to dark and not well-separated titin bands. HT, protein samples from heterozygote RBM20 knockout rat heart; HM, protein samples from homozygote RBM20 knockout rat heart. Titin bands were thick and heavy, and could not be properly distinguished from each other because of high concentration sample induced overstaining.

We use silver staining to detect the protein bands. Silver staining is the most sensitive colorimetric method for detecting total protein. Although the staining is efficient for visualizing low concentration proteins, the high concentration protein bands could be too dark to identify within seconds (Fig. 3). Thus proper dilution of protein sample is essential. For protein samples isolated from ∼15 mg tissue/1 mL buffer, 10 times dilution with sample buffer would be a nice choice based on our experience.

### Sample degradation

**Fig. 4:**
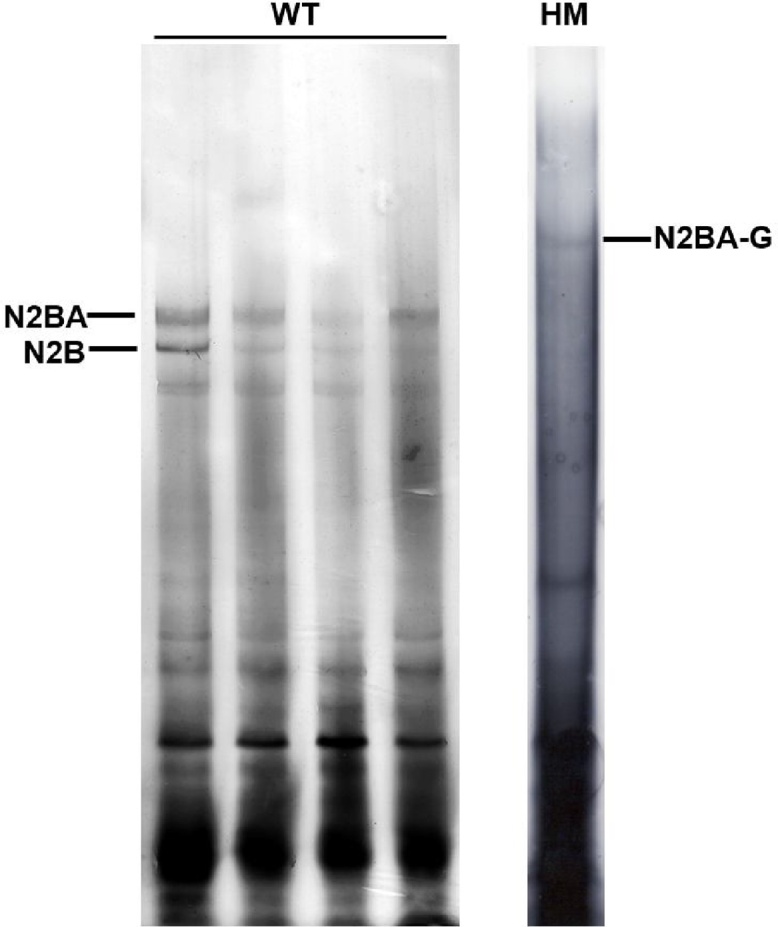
Degraded titin bands. WT, protein sample from heart tissues of wild type rats; HM, protein samples from homozygote RBM20 knockout rats. Titin isoform bands were fuzzy and unidentifiable on the gel because of protein degradation. The degraded proteins also would tend to be smeared on the gel which caused high staining background. In WT, two similar sized N2BA isoform bands could not be detected because of titin protein degradation. In HM, titin band is fuzzy and almost undetectable due to titin protein degradation.

Proper tissue and protein sample storage is important. Titin is the largest protein, and it is very easy to degrade. It happens quite often when other smaller proteins (<0.5 MDa) are clearly and sharply exhibited on the SDS-PAGE gel but the titin bands are smeared on the gel because of degradation (Fig. 4).

### Successful dissolved and separated titin isoforms on the gel

**Fig. 5:**
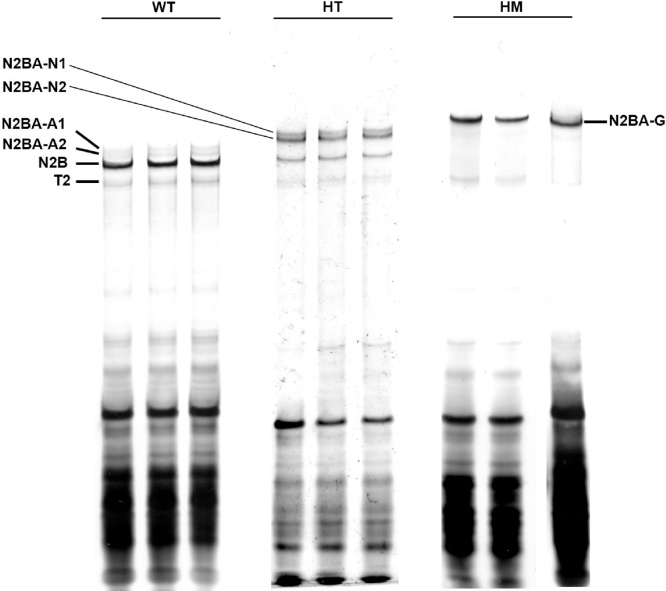
Titin bands of adult rat heart. WT, protein sample from heart tissues of wild type rats; HT, protein samples from heterozygote RBM20 knockout rat heart; HM, protein samples from homozygote RBM20 knockout rats. In WT, three major titin bands (N2BA-A1, A2, and N2B) can be clearly separated and in HT, two slightly larger N2BA isoforms (N2BA-N1 and −N2) and N2B are clear and sharp, while, in HM, the only largest N2BA-G can be detected. T2, a titin proteolytic degradation band.

A nice gel with sharp and clearly separated titin isoform bands can be achieved if all the problems mentioned above can be avoided (Fig. 5).
